# The effect of exogenous calcium on mitochondria, respiratory metabolism enzymes and ion transport in cucumber roots under hypoxia

**DOI:** 10.1038/srep11391

**Published:** 2015-08-25

**Authors:** Lizhong He, Bin Li, Xiaomin Lu, Lingyun Yuan, Yanjuan Yang, Yinghui Yuan, Jing Du, Shirong Guo

**Affiliations:** 1 Key Laboratory of Southern Vegetable Crop Genetic Improvement in Ministry of Agriculture, College of Horticulture, Nanjing Agricultural University, Nanjing 210095, China; 2 Horticulture Research Institute, Shanghai Academy Agricultural Sciences, Key Laboratory of Protected Horticulture Technology, Shanghai, 201403, China; 3 College of Life Science, Anhui Science and Technology University, Fengyang, Anhui 233100, China

## Abstract

Hypoxia induces plant stress, particularly in cucumber plants under hydroponic culture. In plants, calcium is involved in stress signal transmission and growth. The ultimate goal of this study was to shed light on the mechanisms underlying the effects of exogenous calcium on the mitochondrial antioxidant system, the activity of respiratory metabolism enzymes, and ion transport in cucumber (*Cucumis sativus* L. cv. Jinchun No. 2) roots under hypoxic conditions. Our experiments revealed that exogenous calcium reduces the level of reactive oxygen species (ROS) and increases the activity of antioxidant enzymes in mitochondria under hypoxia. Exogenous calcium also enhances the accumulation of enzymes involved in glycolysis and the tricarboxylic acid (TCA) cycle. We utilized fluorescence and ultrastructural cytochemistry methods to observe that exogenous calcium increases the concentrations of Ca^2+^ and K^+^ in root cells by increasing the activity of plasma membrane (PM) H^+^-ATPase and tonoplast H^+^-ATPase and H^+^-PPase. Overall, our results suggest that hypoxic stress has an immediate and substantial effect on roots. Exogenous calcium improves metabolism and ion transport in cucumber roots, thereby increasing hypoxia tolerance in cucumber.

Higher plants frequently experience limited oxygen availability, mainly due to flooding^1^, waterlogging^2^, irrigation or hydroponic culture conditions^3^. Approximately 16% of fertile land worldwide is affected by soil waterlogging^4^. Waterlogging or hydroponic culturing result in lowered levels of oxygen in the plant root zone due to the low diffusion rate of molecular oxygen in water^5^. The consequences of hypoxia, such as a decrease in the cellular energy charge, a drop in cytoplasmic pH, and the accumulation of toxic end products from anaerobic respiration and reactive oxygen species (ROS) during recovery, are responsible for the reduced growth and yield observed in many agriculturally important crops exposed to flooding^6^. Plants subjected to hypoxia undergo dramatic metabolic changes. Defense mechanisms are induced to cope with the potential damage. Cucumber, which is sensitive to oxygen shortage, often faces hypoxic stress, resulting in enormous economic losses.

Oxygen is vital to the central energy-providing pathway of the cell, and the presence or absence of oxygen determines metabolic activity and energy production^7^. Respiration is generally the first aspect of plant metabolism to be affected by oxygen shortage. Hypoxic stress interferes with electron transport chains, and a lack of suitable electron acceptors leads to the saturation of redox chains, accumulation of NAD(P)H and decreased synthesis of ATP^8^. The mitochondrial electron transport chain, with its redox-active electron carriers, is the most likely candidate for the formation of intracellular ROS. ROS production results in damage to physiological metabolism and the cell membrane structure in plants^9^. The metabolic pathways in the mitochondria are also sensitive to environmental changes^10^. Accumulating evidence suggests that the mitochondria may act as ‘sensors’ in the overall plant stress response^11^.

Free Ca^2+^ is one of the key signal molecules in plants and animals and is involved in multiple signal transduction pathways, which are fundamental for many intercellular and intracellular interactions^12^,^13^. Intracellular Ca^2+^ signals are realized by spatially and temporally defined changes in the free Ca^2+^ concentration in the cytosol^14^. Environmental stimuli, such as salt stress^15^, hypoxia^16^, and chilling^17^, can alter the cellular Ca^2+^ concentration. Moreover, the involvement of calcium in hypoxia responses has been observed in many plants. For example, anoxia stress in the cells of maize, rice and wheat plants causes a rise in the cytoplasmic Ca^2+^ concentration^18^. Elevated calcium levels significantly influence the antioxidant system and nitrogen metabolism in hypoxia-stressed muskmelon roots^19^. The measurement of changes in free Ca^2+^ activity in the cytoplasm is crucial for the evaluation of the role of calcium in the transduction of external stimuli in cellular processes^20^.

As ATP generation by oxidative phosphorylation begins to diminish due to O_2_ limitation, an energetic deficit can be overcome by the activation of the anaerobic ATP supply. The reduction in ATP availability has major repercussions for root development, root nutrient uptake and root maintenance. Ion transport ATPases are a major ATP sink in plants cells and tissues^21^. When oxygen is in short supply, these energy-consuming processes affect cell metabolism and the overall plant nutritional status in response to stress. A previous study showed that both hypoxia and anoxia rapidly depolarize the plasma membrane (PM) of higher plants by approximately 50 mV, presumably by inhibiting electrogenic H^+^ pumps^22^. It remains to be determined whether all ion transporters are affected to the same extent by hypoxia. The spatial profile of ions in root tissues is also unknown^23^.

## Results

### The growth conditions of cucumber plants

**Figure 1:**
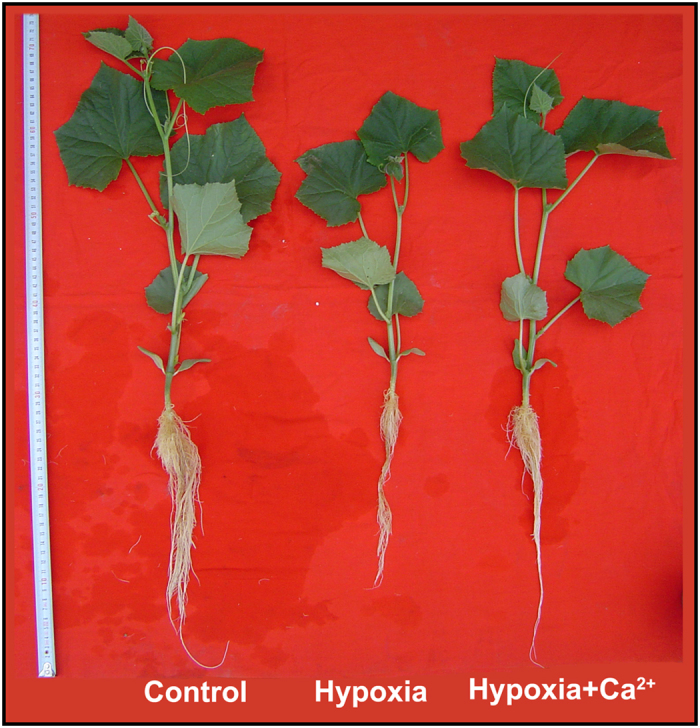
Phenotypes of cucumber seedlings exposed to hypoxia and hypoxia + 4 mM CaCl_2_ treatment for 6 d compared to plants exposed to normoxia conditions (Control). The photographs show plants representative of the entire population.

The effect of hypoxia on cucumber plants was determined by visual observation. After 6 d of treatment, several morphological changes were observed in the stressed plants (Fig. 1). The roots of the hypoxia-treated plants were less dense and more stunted than control roots; hypoxia + calcium treatment reversed this condition. Plants receiving exogenous calcium also exhibited enhanced aboveground growth compared to hypoxia-treated plants, including less etiolated stems, larger leaf areas and an increased number of leaves. Our previous research also revealed that exogenous calcium enhanced the biomass and soluble protein content of cucumber seedlings under hypoxia^24^. Apparently, the growth of cucumber seedlings was significantly hindered. This effect could be partially rescued by the application of exogenous calcium.

### The content of lipid peroxides, free radical production and antioxidant enzyme activity in mitochondria

**Figure 2:**
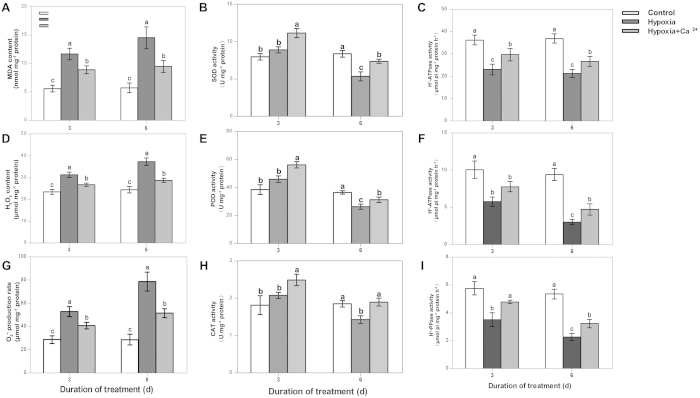
Determination of changes in O_2_^•-^ production and MDA and H_2_O_2_ content (A); the activities of SOD, POD, and CAT (B); plasma membrane (PM) H^+^-ATPase; and tonoplast H^+^-ATPase and H^+^-PPase in the mitochondria of cucumber roots at 3 d and 6 d after treatment. Samples were analyzed under normoxic conditions (Control), hypoxia treatment (Hypoxia) and hypoxia + 4 mM CaCl_2_ treatment (Hypoxia + Ca^2+^). Values are means ± SE of three independent experiments. Bars marked with dissimilar letters are significantly different according to Duncan’s multiple range tests (P < 0.05).

At 3 d and 6 d, the MDA content, H_2_O_2_ content and O_2_^•-^ production rate in the mitochondria of cucumber roots significantly increased under hypoxia. The values of these components increased with increasing treatment times (Fig. 2A,D,G). Treatment with exogenous calcium resulted in a 34.6% reduction in the O_2_^•-^ production rate, a 23.1% reduction in the H_2_O_2_ content and a 34.8% reduction in the MDA content compared to hypoxia-stressed plants at 6 d.

Regarding antioxidant enzyme activities in mitochondria, the activities of SOD, POD and CAT slightly increased at 3 d under hypoxia treatment; however, these increases were not significant. The activities of these enzymes were decreased at 6 d under hypoxia treatment in comparison with the control (Fig. 2B,E,H). Conversely, hypoxia + CaCl_2_ treatment increased the content of these enzymes at both 3 d and 6 d compared to hypoxia-treated plants. At 6 d under treatment, the activity of SOD and POD under both hypoxia and hypoxia + CaCl_2_ treatments was lower than that in control plants. However, the extent of this decline in plants receiving the hypoxia + CaCl_2_ treatment was less than that observed in hypoxia-treated plants. The activity of CAT under hypoxia + CaCl_2_ was not significantly different compared to the control at 6 d.

### The activity of tonoplast H^+^-ATPase and H^+^-PPase and plasma membrane H^+^-ATPase in cucumber roots

A decrease in the activities of PM H^+^-ATPase and tonoplast H^+^-ATPase (Fig. 2C,F) was observed in comparison with the control at 3 d and 6 d. In plants treated with exogenous calcium, the activity of the PM H^+^-ATPase and the tonoplast H^+^-ATPase increased by 28.9% and 25.3%, respectively, compared to the hypoxia-stressed plants at 3 d. At 6 d, the activity of PM H^+^-ATPase and tonoplast H^+^-ATPase in plants treated with exogenous calcium increased by 34.6% and 55.9%, respectively, compared to hypoxia-stressed plants. Nevertheless, the activity of these enzymes did not reach levels observed in control plants. The activity of tonoplast H^+^-PPase (Fig. 2I) significantly decreased under hypoxic conditions compared to the control. After applying exogenous calcium, the activity of this enzyme increased.

### Enzymes involved in glycolysis and tricarboxylic acid metabolism

**Figure 3:**
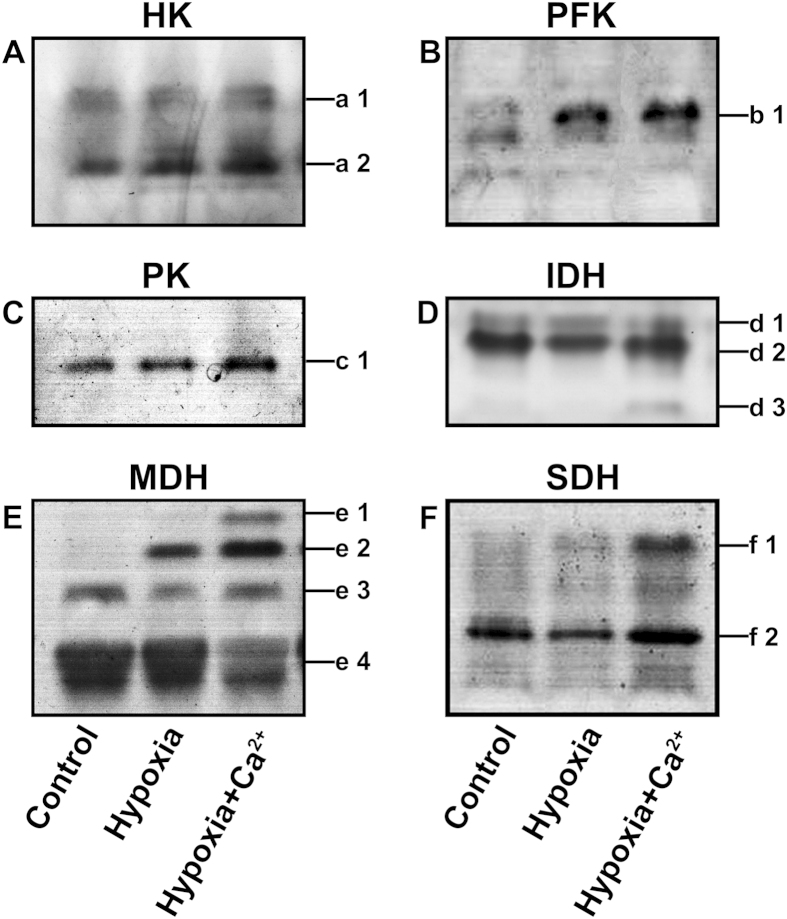
Expression of hexokinase (HK, **A**), 6-phosphofructokinase (PFK, **B**), pyruvate kinase (PK, **C**), isocitrate dehydrogenase (IDH, **D**), malate dehydrogenase (MDH, **E**) and succinate dehydrogenase (SDH, **F**) isozymes in cucumber roots exposed to one of three treatments for 3 d. These three treatments were normoxic conditions (Control), hypoxia treatment (Hypoxia) and hypoxia + 4 mM CaCl_2_ treatment (Hypoxia + Ca^2+^). Lines indicate the isozymes detected by staining.

The enzymes that catalyze the key steps in glycolysis metabolism, such as hexokinase (HK), 6-phosphofructokinase (PFK) and pyruvate kinase (PK), were analyzed. To determine HK activity (Fig. 3A), we measured the levels of two isozymes in all treatments. Under hypoxic conditions, the expression of a1 and a2 were enhanced with respect to the control. Under hypoxia + CaCl_2_ treatment, the expression of HK was further enhanced. With respect to the accumulation of PFK (Fig. 3B) and PK (Fig. 3C), an increase in the expression of these enzymes was observed under hypoxic conditions; hypoxia + CaCl_2_ further enhanced their expression.

Key enzymes involved in tricarboxylic acid metabolism were analyzed, including isocitrate dehydrogenase (IDH), malate dehydrogenase (MDH), and succinate dehydrogenase (SDH). Three IDH isozymes—d1, d2 and d3—were isolated from cucumber roots in all treatments (Fig. 3D). The three treatments had little effect on the expression of d1. However, the accumulation of d2 decreased remarkably under hypoxic stress. In plants treated with hypoxia + CaCl_2_, the expression of the d2 isozyme increased and a new band (d3) was observed. A new MDH band (e2) was observed under hypoxic conditions (Fig. 3E). Two new bands (e1 and e2) were observed under hypoxia + CaCl_2_ conditions; this result suggests that the isozymes e1 and e2 are sensitive to hypoxia and calcium, respectively. The expression of the SDH isozyme f1 was significantly enhanced under hypoxia + CaCl_2_ conditions and decreased in the control and in plants under hypoxic conditions (Fig. 3F). The f2 band was visually weak under the hypoxia treatment compared to the control, whereas exogenous calcium application increased its expression markedly.

**Figure 4:**
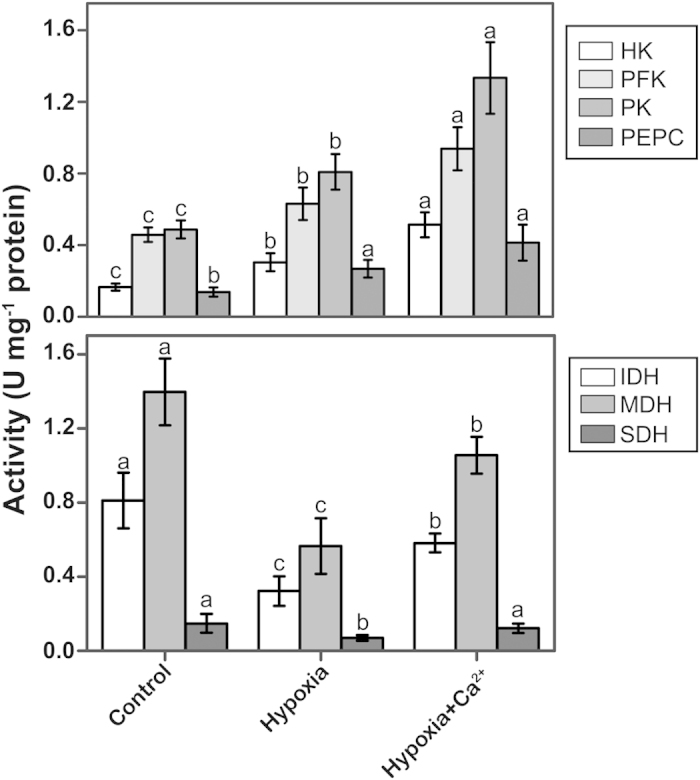
Changes in enzyme activities in cucumber roots at 3 d after treatment. The activities of hexokinase (HK), 6-phosphofructokinase (6-PFK), pyruvate kinase (PK), phosphoenolpyruvate carboxylase (PEPC), isocitrate dehydrogenase (IDH), malate dehydrogenase (MDH) and succinate dehydrogenase (SDH) were determined in root material collected from cucumber plants grown under normoxic conditions (Control), hypoxia treatment (Hypoxia) and hypoxia + 4 mM CaCl_2_ treatment (Hypoxia + Ca^2+^). Values are means ± SE of three independent experiments. Bars marked with dissimilar letters are significantly different from each other according to Duncan’s multiple range tests (P < 0.05).

We also quantified the activities of these enzymes in spectrophotometric assays to reinforce the findings described above (Fig. 4). The activities of HK, PFK, PK and PEPC were observed to increase under hypoxic conditions compared to the control. After applying exogenous calcium, the activities of IDH, MDH and SDH increased further to reach significant levels. Conversely, the activities of IDH, MDH and SDH decreased under hypoxia treatment. Hypoxia + CaCl_2_ treatment increased the activity of these enzymes. Except for SDH, the activities of these enzymes did not reach the levels observed in the control.

### Fluorescence measurements and subcellular localization of Ca^2+^ in the root apex of cucumber

**Figure 5:**
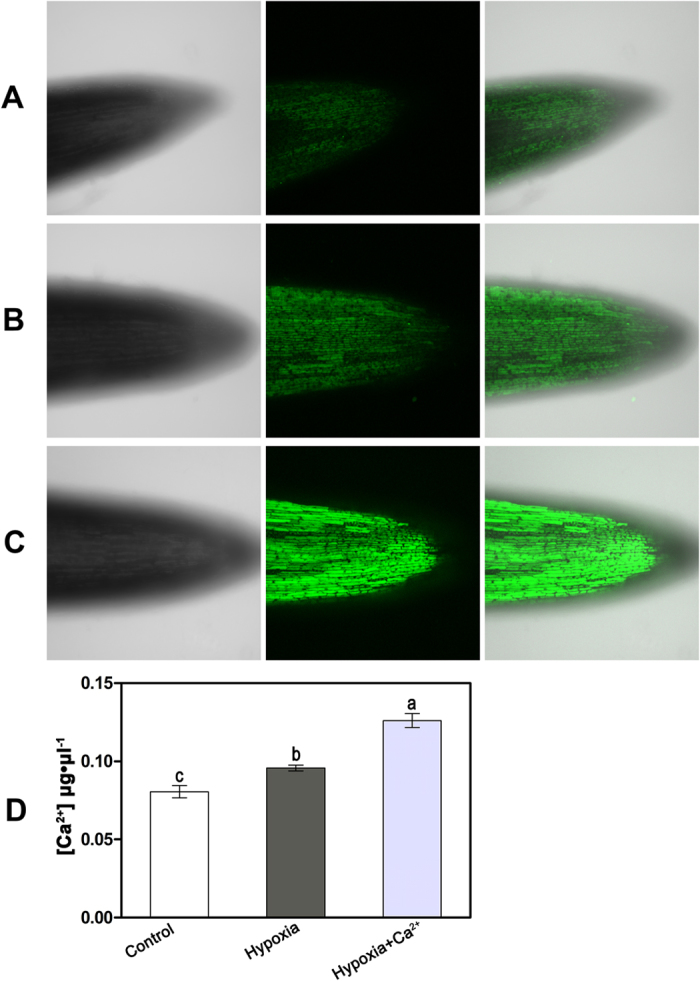
Detection of Ca^2 +^ concertrations in cucumber roots tips at 3 d after treatment. (**A–C**) Confocal images of cucumber roots tips by Fluo-3 AM. (**A**) Cucumber plants grown under normoxic conditions (Control). (**B**) Cucumber plants grown under hypoxic conditions (Hypoxia). (**C**) Cucumber plants grown under hypoxia + 4 mM CaCl_2_ treatment (Hypoxia + CaCl_2_). The signal corresponding to the incorporated Fluo-3 is green. (**D**) The Ca^2+^ content (μg•μl^−1^) of cucumber roots under normoxic conditions (control), hypoxic conditions (Hypoxia) and hypoxia + 4 mM CaCl_2_ treatment (Hypoxia + Ca^2+^). Values are means ± SE of three independent experiments. Bars marked with dissimilar letters are significantly different from each other according to Duncan’s multiple range tests (P < 0.05).

To trace the dynamics of free calcium ions in cucumber roots, the fluorescent indicator Ca^2+^ green 5N/AM was used for loading in the root tips. Detailed analysis under a confocal microscope revealed significant differences between the signal in the treated plants (hypoxia and hypoxia + CaCl_2_) and the control. After loading with Ca^2+^ green 5N/AM, green fluorescence was observed in the middle of the root tips in control plants; most fluorescence occurred in the elongation zone. The meristem region of control plants was practically devoid of green fluorescence (Fig. 5A). Under hypoxia treatment, the region of green fluorescence was more intense compared to the control (Fig. 5B). The greatest range and intensity of the green fluorescence signal were observed under the hypoxia + CaCl_2_ treatment. In plants that received this treatment, the whole root tip exhibited intense green labeling, including the elongation zone, the meristem region and the edge of the root tip (Fig. 5C). The Ca^2+^ content was quantified using the Calcium Colorimetric Assay Kit (BioVision, Mountain View, CA); the quantitative results revealed similar trends (Fig. 5D).

**Figure 6:**
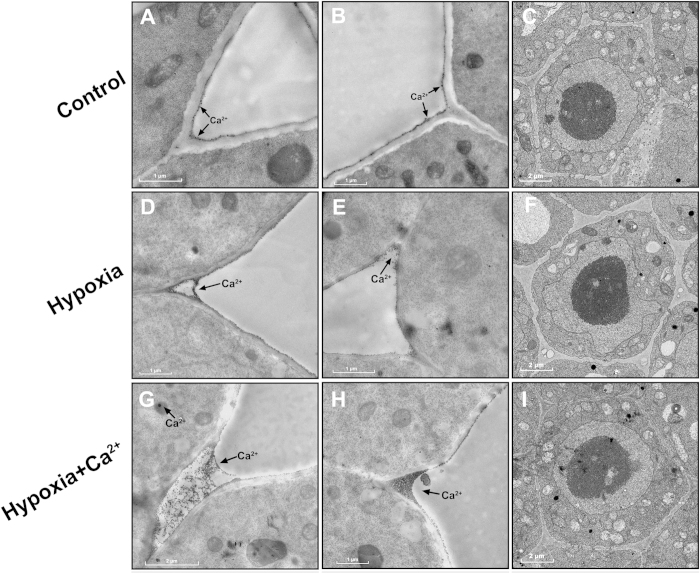
Subcellular localization of Ca^2+^ (A, B, D, E, G, H, J and K) and subcellular distribution (C, F, I and L) in cucumber roots at 3 d after treatment. Samples comprised 3 mm of the root tips. (**A–C**) Cucumber plants grown under normoxic conditions (Control). (**D–F**) Cucumber plants grown under hypoxic conditions. (**G–I**) Cucumber plants grown under hypoxia + 4 mM CaCl_2_ treatment (Hypoxia + Ca^2+^). (**J–L**) Cucumber plants grown under hypoxia + 50 μM LaCl_3_ treatment (Hypoxia + La^3+^). Arrows indicate Ca^2+^ ion precipitates in the root cells.

To study the subcellular distribution of the Ca^2+^ ions, we used the cytochemical method. This method is used to localize free and loosely bound calcium. Precipitates were mainly localized to the plasma membrane and in intercellular spaces. In the control, a few Ca^2+^ deposits were found in the plasma membrane (Fig. 6A,B). The Ca^2+^ level increased in the plasma membrane and in the intercellular spaces after 3 d of hypoxia (Fig. 6D,E). The karyotheca appeared to have contracted and presented with a wavy shape. The number of the mitochondria decreased compared to the control (Fig. 6F). In hypoxia + CaCl_2_-treated samples, a rich pool of fine and dense Ca^2+^ precipitates were localized in the plasma membrane and intercellular spaces; a few Ca^2+^ precipitates were also observed in the cytosol and mitochondria (Fig. 6G,H). The application of exogenous calcium alleviated these changes in the nucleus; more mitochondria were observed compared to in hypoxia-treated samples (Fig. 6I).

### X-ray microanalysis of transverse sections of cucumber roots for [K^+^] and [Ca^2+^]

**Figure 7:**
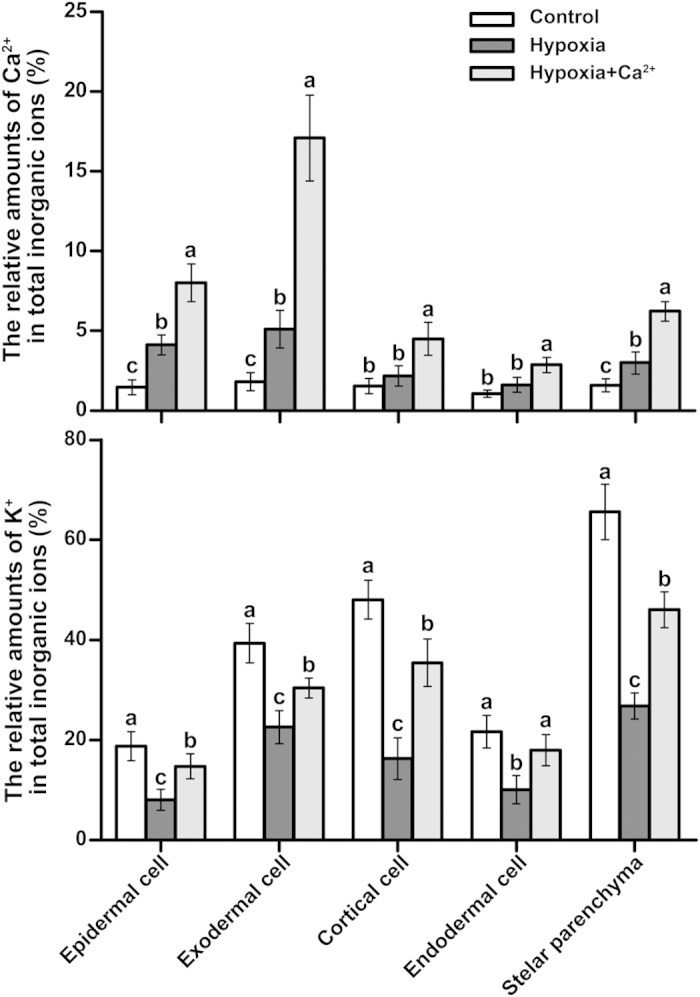
Ca^2+^ and K^+^ level in five different tissues (epidermal cells, exodermis cells, cortical cells, endodermal cell, and the stelar parenchyma) of cucumber roots detected using point-scanning of an X-ray. The plants were grown under normoxic conditions (Control), hypoxic conditions (Hypoxia) and hypoxia + 4 mM CaCl_2_ treatment (Hypoxia + Ca^2+^) for 3 d. Samples comprised 1 cm of the root tips. Values are means ± SE of three independent experiments. Bars marked with dissimilar letters are significantly different from each other according to Duncan’s multiple range tests (P < 0.05).

**Figure 8:**
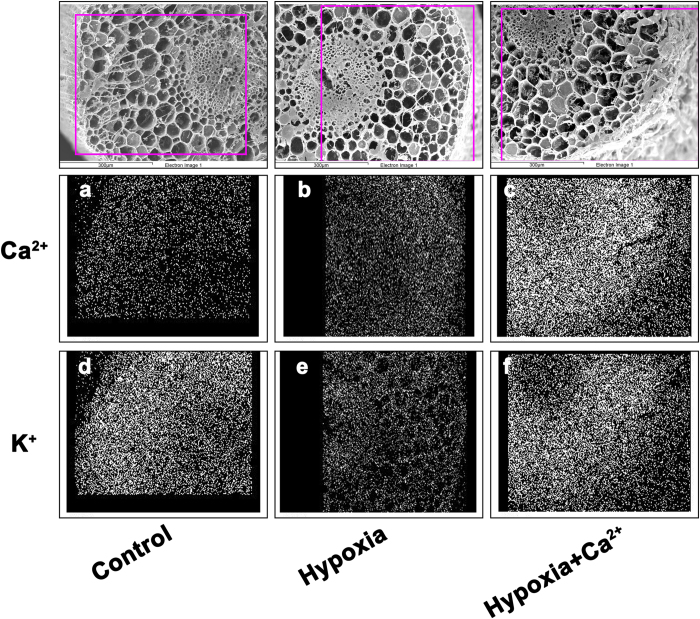
Images of Ca^2+^ and K^+^ distribution in cucumber roots detected using map-scanning of an X-ray. The plants were grown under normoxic conditions (Control), hypoxic conditions (Hypoxia) and hypoxia + 4 mM CaCl_2_ treatment (Hypoxia + Ca^2+^) for 3 d. Samples comprised 1 cm of the root tips. Denser points indicate higher ion concentrations.

Transverse sections of the roots of cucumber seedlings were scanned using X-ray microanalysis. Sectioning began with the outermost tissues and proceeded inward in the following order: epidermal cells, exodermal cells, cortical cells, endodermal cells, and the stelar parenchyma. All of these spectra were transformed into data using professional software supplied with the S-3000N scanning electron microscope. Relative amounts of [K^+^] and [Ca^2+^] in the samples comprising the three treatments in five tissues are shown in Fig 7. In the roots of hypoxia-treated plants, Ca^2+^ levels increased in the epidermal cells, exodermal cells and the stelar parenchyma compared with the control. After applying exogenous calcium, [Ca^2+^] significantly increased at the outer root tissues and toward the middle tissues; such an increase was especially evident in epidermal and exodermal cells. Conversely, [K^+^] greatly decreased under hypoxic stress and increased under the hypoxia + Ca^2+^ treatment (Fig. 7). Exodermal cells, cortical cells and the stelar parenchyma exhibited higher levels of K^+^; both [K^+^] and [Ca^2+^] were asymmetrically distributed. Images of the distrobution of Ca^2+^ and K^+^ in cucumber roots were obtained by map-scanning an X-ray and showed a similar trend (Fig. 8).

## Discussion

To date, there have been few reports concerning the interplay among exogenous calcium, mitochondrial function and respiratory metabolism. However, a previous study revealed that calcium is involved in short-term hypoxic tolerance in muskmelons by promoting NO_3_^−^ uptake and accelerating its transformation into amino acids, heat-stable proteins or polyamines as well as by preventing polyamine degradation^19^. In the present study, cucumber seedlings were shown to be tolerant to hypoxic stress after exogenous calcium was applied (Fig. 1). Growth inhibition is a common phenomenon in plants under hypoxia. This phenomenon has also been observed in waterlogged *Lotus japonicas*^25^. These morphological changes can be explained as part of the adaptive response of plants to hypoxia; plants limit their energy demand and respiratory oxygen consumption by down-regulating the synthesis of assimilate products such as starch and protein^24^. If the demand for respiratory oxygen consumption decreases, anoxia in plant tissues can be postponed or even prevented^26^.

The resistance to stress and the response to a variety of stress signals are influenced by the mitochondria status in plants^27^. The role of calcium in the mitochondria under hypoxic conditions was discussed in a review by Stael *et al.*^28^. The root apoplast is another important site for ROS generation and scavenging. Central to ROS signaling is the induction of Ca^2+^ influx across the plasma membrane. ROS such as ^•^OH can also stimulate both the outward K^+^ current and hyperpolarization-activated Ca^2+^-permeable channels^29^. The production and detoxification of ROS likely play widespread roles in signaling and damage at the onset of and release from low-oxygen stress^30^. The major ROS-scavenging enzymes in plants include SOD, POD, CAT, APX, glutathione peroxidase and peroxiredoxin^31^. A recent study demonstrated that the Arabidopsis PPR40 protein is associated with the mitochondrial inner membrane complex III, and its malfunction results in ROS accumulation, SOD activation, lipid peroxidation and the altered expression of stress-responsive genes^32^. A study based on the expression of a peroxisome-targeted chameleon probe (a Ca^2+^ reporter protein construct) revealed that plant peroxisomes undergo Ca^2+^ fluxes^33^. In the present study, low oxygen also enhanced ROS levels in the mitochondria of cucumber roots (Fig. 2). When CaCl_2_ was applied, the ROS content in mitochondria decreased compared to that under the hypoxia treatment at both 3 d and 6 d. This result suggests that exogenous calcium decreases lipid peroxidation by alleviating the increase in ROS levels under hypoxic stress. A decline in the activity of antioxidant enzymes was observed at 6 d in hypoxia-treated samples compared to the control (Fig. 2). This finding indicates that a longer period of hypoxic stress destroyed the antioxidant system in mitochondria. Exogenous calcium enhanced hypoxic tolerance in cucumber plants by promoting the activity of antioxidant enzymes and decreasing ROS production in mitochondria. Similar results were also obtained in the roots of cucumber seedlings under hypoxic stress^34^ and in eggplant seedlings under chilling^17^.

An alteration in the isozymic pattern usually occurs under abiotic stress^35^. Antioxidant isozymes, glutamate dehydrogenase isozymes and many other isozymes have been analyzed by gel electrophoresis in previous studies^36^. However, the key isoenzymatic patterns of the glycolytic and tricarboxylic acid (TCA) cycle have rarely been reported. Moore *et al.*^37^ observed that Arabidopsis plants use a specific hexokinase (HXK1) as a glucose sensor to interrelate nutrient, light, and hormone signaling networks to control growth and development in response to a changing environment. PFK is a major enzyme that controls glycolytic flux and is likely a rate-limiting enzyme of glycolysis under anoxia^38^. The activity of PFK increased in peach fruits in response to anoxia^39^. In our experiments, all of the tested enzymes involving glycolytic metabolism were up-regulated under hypoxia (Figs 3,4). This result suggests that the glycolytic pathway, a major source of energy, was up-regulated under anoxic circumstances^24^. Increased glycolytic flux is also needed to support pyruvate demand in the fermentative pathways induced by anoxia^39^. After applying CaCl_2_, the expression and activity of most enzymes, including HK, PFK, and PK, increased compared to those in hypoxia-treated samples. These results indicated that exogenous calcium further increases the activity of glycolytic enzymes to increase the rate of glycolytic flux^40^, thereby increasing the tolerance of cucumber seedlings to hypoxia. The TCA cycle is a fundamental component of mitochondrial respiration and links glycolysis and/or extramitochondrial malate synthesis to the mitochondrial electron transport chain. As part of an ongoing project, a wide range of transgenics and mutants deficient in the expression of TCA cycle enzymes were analyzed. Growing plants deficient in the expression of mitochondrial MDH under short-day conditions resulted in a dwarf phenotype^41^. In a previous study, the activities of IDH, MDH and SDH decreased in *Lotus japonicas* under waterlogging^24^. Moreover, calcium acts to maintain higher MDH and SDH activities and a certain level of aerobic respiration in pepper^42^. The accumulation and activity of IDH, MDH and SDH were all induced by exogenous calcium (Figs 3,4). In our experiments, Ca^2+^ positively affected ATP production by up-regulating the expression of major limiting enzymes of the TCA cycle. Such activity enhanced hypoxic tolerance in cucumber.

**Figure 9:**
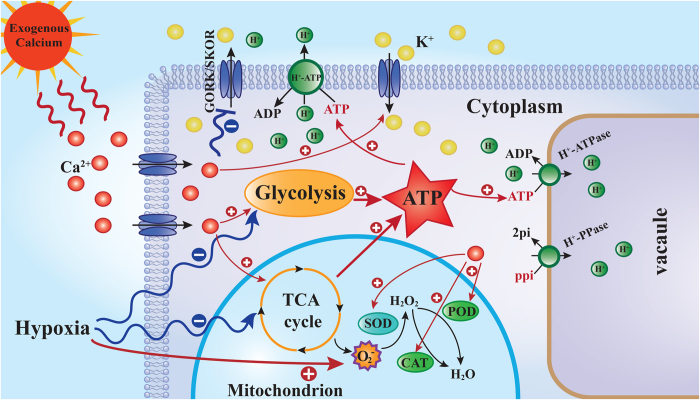
A simplified metabolic scheme and signal transduction pathway for Ca^2 +^ in cucumber under hypoxia. The model is based on the results presented here. The red arrow with a straight line and + symbol indicates up-regulation. The blue arrow with a curve and − symbol indicates down-regulation. GORK: guard cell outward rectifying K^+^ channel; SKOR: stelar K^+^ outward rectifier.

K^+^ fluxes have been extensively evaluated in plants under environmental stresses such as salinity^43^ and hypoxia^23^. As K^+^ uptake is disturbed by oxygen deprivation^21^, an improved K^+^ uptake in roots may be critical to overall plant performance under hypoxia. Latz *et al.*^44^ have shown that Ca^2+^-dependent protein kinases activate the vacuolar K^+^ channel TPK1 under salt stress to maintain higher cytosolic K^+^ levels. Hypoxia also led to depolarization-induced K^+^ efflux via outward-rectifying depolarization-activated PM K^+^ channels (GORK/SKOR)^45^. However, Ca^2+^ is a potent blocker of these channels. Extracellular Ca^2+^ can ameliorate NaCl-induced K + loss in *Arabidopsis* root and leaf cells by controlling PM K + -permeable channels^46^. Our observation that K^+^ concentrations decrease in roots under hypoxia and increase after the application of exogenous Ca^2+^ may indicate that similar mechanisms are also active under hypoxia (Figs 7,8). Our findings are also consistent with previous reports showing that hypoxia significantly inhibits K^+^ uptake in barley roots^23^. Pre-treatment with vanadate, a known inhibitor of the PM H^+^-ATPase, significantly increased net H^+^ in barley roots. This finding supports the hypothesis that a substantial component of H^+^ transport under hypoxia is due to changes in the activity of PM H^+^-ATPase. A decrease in the activity of proton-pumping ATPases not only reduced K^+^ uptake but also led to the acidification of the cytosol under anoxic conditions. Such acidification results from protons released through ATP hydrolysis and from the low ATP concentration, which reduces the activity of the proton pump. It has also been suggested that pyrophosphate (PPi) can substitute for ATP as an energy source, which would represent an important acclimation mechanism in anoxia-tolerant species^47^. For example, the activity of tonoplast H^+^-PPiase increased 75-fold in rice seedlings after 6 d under anoxia^48^. The expression of one gene encoding tonoplast H^+^-PPase (Os02g55890) was up-regulated by 35-fold in anoxic rice coleoptiles^49^. Calcium-binding mitochondrial carrier proteins (APC1, APC2 and APC3) can function as mitochondrial ATP-importers^50^. Mitochondrial ATP import might be a first line of defense against hypoxia, keeping the mitochondrial membrane proton gradient and the electron transport chain intact to prevent ROS generation. In our experiment, we also observed that hypoxia inhibits the activity of PM H^+^-ATPase and tonoplast H^+^-ATPase and slightly induces the activity of tonoplast H^+^-PPase; exogenous calcium increases the activity of these enzymes (Fig. 2). Exogenous calcium resulted in the maintenance of a high K^+^ level via an increase in the activity of enzymes involved in respiratory metabolism; mitochondrial homeostasis was maintained, and sufficient ATP was supplied to these ion transport ATPases (Fig. 9). A high K^+^ level resulted in better ion homeostasis and regulated cell turgor, as K^+^ significantly affects the activity of vacuolar ion channels^51^.

In summary, our results indicate that hypoxia signal transduction pathways are distinct in terms of Ca^2+^ elevation. External Ca^2+^ is involved in hypoxia signaling in cucumber roots. Exogenous calcium, through decreased ROS levels, enhanced the antioxidant system in mitochondria and enhanced mitochondrial homeostasis. Furthermore, exogenous calcium increased the accumulation of respiratory metabolism enzymes and mediated the transport of Ca^2+^ and K^+^ to strengthen hypoxia tolerance in cucumber roots.

## Materials and Methods

### Plant materials and growth conditions

Cucumber (*Cucumis sativus* L. cv. Jinchun No. 2, hypoxia sensitive^52^) seeds were sterilized with 0.5% (W/V) sodium hypochlorite solution for 10 min and then washed thoroughly with deionized water. The washed seeds were sown in two layers of wet filter paper and incubated in the dark at 28 °C for 24 h. The germinated seedlings were transplanted to plastic trays (41 × 41 × 5 cm) containing quartz sand and grown at 25–30 °C (day) and 15–18 °C (night), with 60–75% relative humidity (RH), in a greenhouse at Nanjing Agriculture University in 2012. The seedlings were supplied with half-strength Hoagland nutrient solution (pH 6.5 ±0.1, EC 2.0–2.2 dS m^−1^). When the second leaves were fully expanded, relatively uniform seedlings were transferred to tanks containing half-strength Hoagland nutrient solution. The solution was renewed every 3 d. The solution in the tanks was kept at 20–25 °C and aerated with an air pump at an interval of 20 min to maintain the dissolved oxygen (DO) level at 8.0 ±0.2 mg l^−1^ (the optimum DO level for cucumber). When the 3rd leaf developed, seedlings were subjected to one of the following three treatments: (1) Control: half-strength Hoagland solution (containing 2 mM Ca^2+^) with a DO level of 8.0 ±0.2 mg l^−1^; (2) Hypoxia treatment: half-strength Hoagland solution (containing 2 mM Ca^2+^) with a DO level of 1.0 ±0.1 mg l^−1^, which was prepared by pumping N_2_ gas into the nutrient solutions; the oxygen concentration in the nutrient solutions was monitored with an automatic DO control system (Quantum-25, Quantum Analytical Instruments Inc., USA); (3) Hypoxia + CaCl_2_ treatment: half-strength Hoagland solution +4 mM CaCl_2_ with DO of 1.0 ±0.1 mg l^−1^; the oxygen concentration in the nutrient solutions was controlled as in the hypoxia treatment.

The roots of the control and treated seedlings were sampled after 3 and 6 d of treatment. Tissues were immediately frozen in liquid nitrogen and stored at −80 °C for further experiments.

### Determination of lipid peroxidation, free radical production and antioxidant enzyme activity in mitochondria

Intact mitochondria were isolated using a method described previously with a slight modification^53^,^54^. The levels of lipid peroxides in the mitochondria were determined by measuring the malondialdehyde (MDA) content from the thiobarbituric acid (TBA) reaction as described previously. A 1 ml suspension of mitochondria was added to 2 ml of a solution containing 20% trichloroacetic acid (TCA) and 0.6% TBA. Tubes were placed in a 95 °C water bath for 20 min and then immediately cooled on ice for 10 min. The samples were centrifuged at 8000 × *g* at 4 °C for 15 min. The MDA concentration was calculated using the A_532_ value, and the nonspecific turbidity of the measurements was corrected by subtracting the value at A_600_. MDA values were expressed in nmol MDA/mg protein. The superoxide production rate was measured according to a modified version of the method described previously. The measurement was performed at A_530_. The O_2_^·-^ formation rate was calculated from a standard curve of NaNO_2_ and expressed as nmol/min/mg protein. The H_2_O_2_ content in the mitochondria was based on the absorbance change of the titanium peroxide complex measured at A_415_. The H_2_O_2_ content was expressed as μmol/mg protein.

One unit of superoxide dismutase (SOD, EC 1.15.1.1) activity was defined as the amount of enzyme required to cause 50% inhibition of the photochemical reduction of nitroblue tetrazolium (NBT). The total volume of the assay mixture was 3 ml and contained 50 mM PBS-Na (pH 7.8), 15 mM methionine, 2.25 mM NBT, 60 μM riboflavin, 30 mM EDTA and 0.01 ml of a suspension of mitochondria. The SOD activity was monitored at 560 nm using a spectrophotometer (WFZ ultraviolet/VIS-2600; UNIC, Shanghai, China) and expressed as U/mg protein. The activity of peroxidase (POD, EC 1.11.1.7) was measured as previously described. One unit of activity was defined as the amount of enzyme required to increase the optical density at 470 nm·min^−1^ by 1 absorbance unit. Measurements were expressed as U/mg protein. The catalase (CAT, EC 1.11.1.11) activity was calculated by measuring the disappearance of H_2_O_2_ over 1 min at 240 nm. One unit of activity was defined as the amount of enzyme required to decrease the optical density by 0.1 absorbance units. Measurements were expressed as U/mg protein.

### Native gel electrophoresis for the visualization of isozymes and the assay of enzyme activity

Frozen root samples (0.2 g) were finely ground into a powder in liquid nitrogen and homogenized on ice in 0.6 ml Tris-HCl buffer (0.1 M pH 7.0) containing 15% sucrose, 0.2% β-mercaptoethanol, and 1% PVP. The homogenate was centrifuged at 12,000 × *g* for 20 min at 4 °C. The supernatant was centrifuged a second time under the same conditions for 10 min and used as directed for native polyacrylamide gel electrophoresis (PAGE). The PAGE gel consisted of a 3.1% stacking gel and a 7.5% separating gel. The gel was electroporated at 80 V for approximately 60 min at 4 °C (in a refrigerator) and then at 180 V for approximately 4 h. The staining solutions for each isozyme were as follows^55^:

- (I) Hexokinase (HK, EC 2.7.1.1): 50 ml of a 0.1 M Tris-HCl (pH 7.8) solution containing 100 mg D-glucose, 60 mg ATP-Na_2_, 30 mg NAD, 10 mg MTT, 2 mg PMS, 20 mg MgCl_2_•6H_2_O, and 40 U G-6-PD was used. The gel was soaked in the Tris solution in the dark at 37 °C until dark blue bands were visible. Coloration was then stopped by the addition of 25% ethanol.
- (II) 6-Phosphofructokinase (PFK, EC 2.7.1.11): 50 ml of a 0.1 M Tris-HCl (pH 8.3), solution containing 100 mg ATP-Na2, 80 mg D-fructose-6-phosphate, 70 mg phosphoenolpyruvate, 30 mg NADH, 80 mg MgCl_2_•6H_2_O, 120 mg KCl, 180 U pyruvate kinase, and 250 U lactate dehydrogenase (LDH) was used. The gel was incubated at 37 °C and observed under long-wave ultraviolet light.
- (III) Pyruvate kinase (PK, EC 2.7.1.40): 50 ml of a 0.2 M Tris-HCl (pH 8.0) solution containing 125 mg ADP-Na2, 75 mg phosphoenolpyruvate, 35 mg NADH, 150 mg MgCl_2_•6H_2_O, 200 mg KCl, and 250 U LDH was used. The gel was incubated at 37 °C for 30–60 min and observed under long-wave ultraviolet light.
- (IV) Isocitrate dehydrogenase (IDH, EC 1.1.1.42): 100 ml of a 0.1 M Tris-HCl (pH 8.0) solution containing 3 ml 0.1 M isocitrate, 20 mg NADP, 10 mg NBT, 3 mg PMS, and 0.4 ml 0.25 M MnCl_2_ was used. The gel was soaked in the mixture in the dark at 37 °C until dark blue bands were visible. Coloration was then stopped by the addition of 25% ethanol.
- (V) Malate dehydrogenase (MDH, EC 1.1.1.37): 100 ml of a 0.1 M Tris-HCl (pH 8.0) solution containing 250 mg L-malic acid, 30 mg NAD, 25 mg NBT, and 2 mg PMS was used. The gel was soaked in the mixture in the dark at 37 °C until dark blue bands were visible. Coloration was then stopped by the addition of 25% ethanol.
- (VI) Succinate dehydrogenase (SDH, EC 1.3.99.1): The gel was soaked in mixture A (containing 50 ml 200 mM PBS at pH 7.5, 20 ml 200 mM succinate, 20 ml 10 mM KCN, 5 ml 32.5 mg/ml PMS, and 5 ml 5 mg/ml MTT) and solution B (2% agar solution at 60 °C).

The stained gels were scanned using Image Scanner III software (GE Healthcare, Piscataway, NJ).

The activities of enzymes were measured using enzyme kits. The kits for measuring HK, PFK, PK, PEPC (phosphoenolpyruvate carboxylase), IDH, MDH, SDH and soluble proteins were purchased from Comin Biotechnology Co. Ltd (Suzhou, China), and the manufacturer’s instructions were followed.

### Transmission electron microscope analysis to determine Ca^2+^ localization and subcellular distribution in the cells of root tips

Three-millimeter-long root tip segments were fixed according to the method previously described^56^. The cut root tips were immersed in a solution containing 2% glutaraldehyde, 2.5% paraformaldehyde, 2% potassium pyroantimonate (K[Sb(OH)]_6_) and 0.1% tannic acid in a 0.1 M phosphate buffer (pH 7.6) for 6 h. The materials were rinsed three times with 0.1 M phosphate buffer containing 2% K[Sb(OH)]_6_. Post-fixation was carried out overnight (approximately 16 h) at 4 °C in 1% OsO_4_ and 2% K[Sb(OH)]_6_. After dehydration in an ethanol-acetone series, the materials were embedded in spur resin. Ultrathin sections for electron microscopy were prepared and stained before being examined using a transmission electron microscope (Transmission Electron Microscope H-7650; Hitachi, Japan).

### X-ray microanalysis

Roots of seedlings at the three-leaf stage from different treatments were washed with distilled water three times. Root segments, including the tip and 1 cm or more of the root, were dipped in 5% agar, inserted to a depth of 1 cm in a copper holder, and immediately sliced free-hand with a razor blade to obtain transverse sections; the sections were then frozen in liquid nitrogen^57^. The samples were freeze dried, gold coated in a high-vacuum sputter coater, and stored in a desiccator. The samples were analyzed under a scanning electron microscope (S-3000N Hitachi, Japan) equipped with an energy-dispersive X-ray detector^58^. Five tissues—epidermal cells, exodermal cells, cortical cells, endoderm cells and the stelar parenchyma—from each root section were analyzed. Three transverse sections from each treatment were observed, and three locations within the same tissue in each section were analyzed. Both map scanning and line scanning were performed.

### Laser scanning confocal microscopy analyses of Ca^2+^ concentrations in root tips

To analyze Ca^2+^ levels, cucumber root tips were loaded with the Ca^2+^ indicator Ca^2+^ green 5N/AM (Molecular Probes, Inc.) and incubated at 28 °C for 60 min in the dark. The roots were washed carefully to remove excess fluorophore. Changes in intracellular Ca^2+^ levels were measured using a digital imaging system equipped with a laser confocal scanning microscope (Leica TCS SP2, Germany).

### Quantification of Ca^2+^ content

Ca^2+^ content was measured using the Calcium Colorimetric Assay Kit (BioVision, Mountain View, CA) following the manufacturer’s instructions. The Ca^2+^ content was measured using a TECAN Infinite M200 Pro Reader (TRCAN, Switzerland) and was expressed as the optical density (OD) at 575 nm in μg per well. The final Ca^2+^ measurements were calculated according to the manufacturer’s protocol and were given in μg per μl of sample. A standard curve was prepared using known amounts of the Ca^2+^ standard included in the kit.

### Determination the activities of tonoplast H^+^-ATPase and H^+^-PPase and plasma membrane H^+^-ATPase

With reference to the previous method^59^, the activity of plasma membrane (PM) H^+^-ATPase was measured in a 0.5 ml reaction volume containing 30 mM Hepes-Tris (pH 6.5), 3 mM MgSO_4_, 0.1 mM (NH_4_)MoO_4_, 50 mM KNO_3_, 0.5 mM NaN_3_, and 0.2% Triton X-100. The reaction was initiated by the addition of 50 μl 30 mM Na_2_-ATP and terminated by the addition 50 μl 55% TCA after incubation at 37 °C for 30 min. The solution was incubated at room temperature for 15 min; 2.5 ml of inorganic phosphorus protection agent containing 16 mM EDTA-Na2, 4% (NH_4_)MoO_4_, 1 mM PVP, 172 mM (H_2_NOH)_2_H_2_SO_4_, 87.5 mM H_2_SO_4_ and 0.25 ml 6.47 mM NaOH was then added. After letting the reaction continue for 40 min, we measured the OD at 660 nm using a spectrophotometer. The activity of the plasma membrane H^+^-ATPase was expressed as Pi•mg^−1^ protein•h^−1^. The method used to measure the activity of tonoplast H^+^-ATPase was similar to that used to measure the activity of plasma membrane H^+^-ATPase. A 0.5 ml aliquot of reaction buffer contained 30 mM Hepes-Tris (pH 7.5), 50 mM KCl, 3 mM MgSO_4_, 0.1 mM (NH_4_)_2_MoO_4_, 0.1 mM Na_3_VO_4_, and 0.5 mM NaN_3_. The reaction was started by adding 50 μl of 30 mM ATP-Na_2_. The remaining steps were identical to the method used to measure the activity of the plasma membrane H^+^-ATPase. The method used to measure the activity of the tonoplast H^+^-PPase was also identical to the method used to measure the plasma membrane H^+^-ATPase, except that the reaction buffer contained 30 mM Hepes-Tris (pH 8.0) and 50 μl 3 M Na_4_PPi was used to start the reaction^60^.

## Additional Information

**How to cite this article**: He, L. *et al.* The effect of exogenous calcium on mitochondria, respiratory metabolism enzymes and ion transport in cucumber roots under hypoxia. *Sci. Rep.* **5**, 11391; doi: 10.1038/srep11391 (2015).
